# Shear Stress Ameliorates Superoxide Impairment to Erythrocyte Deformability With Concurrent Nitric Oxide Synthase Activation

**DOI:** 10.3389/fphys.2019.00036

**Published:** 2019-02-05

**Authors:** Lennart Kuck, Marijke Grau, Wilhelm Bloch, Michael J. Simmonds

**Affiliations:** ^1^Biorheology Research Laboratory, Menzies Health Institute Queensland, Griffith University, Gold Coast, QLD, Australia; ^2^Department of Molecular and Cellular Sport Medicine, German Sport University Cologne, Cologne, Germany

**Keywords:** hemorheology, shear conditioning, antioxidants, oxidative stress, cell deformability

## Abstract

The cellular deformability of red blood cells (RBC) is exceptional among mammalian cells and facilitates nutrient delivery throughout the microcirculation; however, this physical property is negatively impacted by oxidative stress. It remains unresolved whether the molecular determinants of cellular deformability – which in the contemporary model of RBC are increasingly recognized – are sensitive to free radicals. Moreover, given cellular deformability has recently been demonstrated to increase following exposure to specific doses of mechanical stimulation, the potential for using shear “conditioning” as a novel method to reverse free-radical induced impairment of cell mechanics is of interest. We thus designed a series of experiments that explored the effects of intracellular superoxide (O_2_^-^) generation on the deformability of RBC and also activation of pivotal molecular pathways known to regulate cell mechanics – i.e., PI3K/Akt kinase and RBC nitric oxide synthase (NOS). In addition, RBC exposed to O_2_^-^ were conditioned with specific shear stresses, prior to evaluation of cellular deformability and activation of PI3K/Akt kinase and RBC-NOS. Intracellular generation of O_2_^-^ decreased phosphorylation of RBC-NOS at its primary activation site (Ser^1177^) (p < 0.001), while phosphorylation of Akt kinase at its active residue (Ser^473^) was also diminished (p < 0.001). Inactivation of these enzymes following O_2_^-^ exposure occurred in tandem with decreased RBC deformability. Shear conditioning significantly improved cellular deformability, even in RBC previously exposed to O_2_^-^. The improvement in cellular deformability may have been the result of enhanced molecular signaling, given RBC-NOS phosphorylation in RBC exposed to O_2_^-^ was restored following shear conditioning. Impaired RBC deformability induced by intracellular O_2_^-^ may be due, in part, to impaired activation of PI3K/Akt, and downstream signaling with RBC-NOS. These findings may shed light on improved circulatory health with targeted promotion of blood flow (e.g., exercise training), and may prove fruitful in future development of blood-contacting devices.

## Introduction

The physical properties of blood are key determinants of flow within the circulatory system. At the macrocirculatory scale, viscosity of plasma and blood are primary determinants of fluidity, while at the smaller scale of the microcirculation, the mechanical properties of red blood cells (RBC) become increasingly important for tissue flux (Chien, 1987). This is particularly evident when considering the RBC (∼8 μm) must traverse apertures less than half its resting diameter (2–3 μm) throughout the capillary network (Diez-Silva et al., 2010). The unique capacity of RBC to deform under shear is related to distinct cell properties (for extensive review, see Baskurt and Meiselman, 2003). The geometry of RBC, for example, is characterized by a biconcave-discoid shape that provides excess surface area relative to cell volume, thereby enabling deformation without expanding surface area. While the RBC membrane exhibits visco-elastic properties owed to the organization of its skeletal protein network, cytosolic viscosity is determined by intracellular hemoglobin concentration. Although RBC deformability had long been attributed to such passive determinants, active regulation of RBC deformability has been proposed (Bor-Kucukatay et al., 2003; Grau et al., 2013; Meram et al., 2013).

Evidence for active regulation of RBC deformability largely stem from studies exploring cellular responses to prior shear stress exposure (i.e., shear “conditioning”). Meram et al. (Meram et al., 2013) reported that when RBC suspensions were exposed to prolonged shear stress within (or just above) the physiological range (5∼20 Pa), cell deformability was unexpectedly increased. Others explored this response, and demonstrated that the findings were repeatable, irrespective of whether shear exposure was continuous or intermittent (Simmonds et al., 2014). These findings were highly specific, however, given it was also reported that once shear stress was 2–3 times greater than the upper limits of the physiological range, impairments in cell deformability were detected, thus indicating damaging effects of excessive shear conditioning (Simmonds and Meiselman, 2016). It remains unknown how active modulation (i.e., shear-mediated changes) of RBC deformation is regulated, although the involvement of free radicals has been postulated. Indeed, both improvements and impairments in RBC deformability have been linked to nitric oxide (NO) and superoxide (O_2_^-^), respectively (Baskurt et al., 1998; Bor-Kucukatay et al., 2003).

It is now widely accepted that RBC express a functional endothelial-type NO synthase (i.e., RBC-NOS), and its activation state is amplified following the cell being exposed to shear stress (Kleinbongard et al., 2006). Activation of RBC-NOS is associated with increased production of NO and augmentation of RBC deformability (Bor-Kucukatay et al., 2003; Ulker et al., 2009, 2011). The shear-mediated improvement in RBC deformability is not well-understood, despite being observed by multiple groups. The working hypothesis is that upon shear stress exposure, mild calcium flux into RBC via mechanosensitive channels (e.g., piezo1) facilitates activation of several proteins, including RBC-NOS (Ulker et al., 2011). Specifically, shear stress appears to stimulate the PI3K/Akt kinase pathway, and thus also downstream enzymes including RBC-NOS (Suhr et al., 2012); activation of RBC-NOS may be regulated by phosphorylation of its serine residue 1177 (Ser^1177^). The associated improvement in cell mechanics appears related to the specific target of NO produced by this pathway: NO may induce post-translational changes to the cysteine groups of integral membrane proteins. That is, S-nitrosylation of α- and β-spectrins has been reported following RBC-NOS activation, and is thought to be critically involved in the ability for RBC to deform (Grau et al., 2013). There is potential for negative perturbations due to excess NO production, however, given unbound NO reacts rapidly with several partners due to its redox properties, including O_2_^-^.

Superoxide is a ubiquitous free radical that is produced constantly during multiple enzymatic processes, including the turnover of oxygen, and the development of muscle force (Bedard and Krause, 2007; Powers and Jackson, 2008). Elevated levels of O_2_^-^ are well-known to impair RBC deformability (Simmonds et al., 2011), most likely due to cross-linking within membrane proteins and/or between hemoglobin and membrane proteins (Baskurt et al., 1998). Whether O_2_^-^ has an effect on the signaling pathways within RBC, and thus also the “active” regulation of cellular deformability, remain unresolved.

It is plausible that O_2_^-^ may diminish NO-mediated improvements, or promote impairment, in cell deformability given that O_2_^-^ readily reacts with NO to form peroxynitrite (ONOO^-^). This reduction in intracellular NO concentration would plausibly decrease the amount of S-nitrosylation of membrane proteins, and thus diminish NO-mediated increases in cell deformability. Moreover, accumulation of ONOO^-^ is reported to alter ion-pump function and organization of the RBC membrane (Starodubtseva et al., 2008), and is associated with multiple pathological processes typical of rheological impairment including vascular disease, ischemia-reperfusion injury, circulatory shock, and inflammation (Szabo et al., 2007).

The potential to exploit mechanical processes to facilitate enhanced RBC deformability is attractive for therapeutic purposes; however, it is unclear whether shear-mediated improvements in cellular deformability (plausibly induced by increased NO generation) remain effective in the presence of O_2_^-^. Moreover, the specific intracellular processes involved in O_2_^-^ mediated impairment of RBC deformability remain poorly understood. Consequently, a series of experiments were designed to explore: (i) the effects of elevated intracellular O_2_^-^ concentration on PI3K/Akt and RBC-NOS activation, and cellular deformability of RBC; (ii) whether mechanical stimulation of RBC, using shear conditioning known to augment cellular deformability, might ameliorate the impaired cell deformability induced by O_2_^-^. In the case that mechanical stimulation was able to reverse the O_2_^-^ induced impairment of cell deformability, whether activation levels of RBC-NOS could explain this response was also examined.

## Materials and Methods

### Selection of Participants and Blood Sample Collection

All participants were healthy males (n = 14; age: 27 ± 5 y; height: 179 ± 8 cm; body mass 77 ± 11 kg) with no known cardiovascular, respiratory, or endocrine pathologies. Participants did not report use of any medication or any history of regular cigarette smoking. The risks and benefits of the study were explained to the participants, before written and witnessed consent was obtained. Blood was collected from a prominent antecubital vein by an accredited phlebotomist. Blood was transferred into a tube coated with ethylenediaminetetraacetic acid (1.8 mg⋅mL^-1^) within 90 s of tourniquet application. All experimental procedures were completed within 4 h of blood collection and each participant’s blood was used once in this study; where necessary (e.g., molecular studies), immediate analyses and/or fixing procedures were implemented. The protocols of the present study were reviewed and approved by the Human Research Ethics Committee (Griffith University, Australia) and are consistent with The Code of Ethics of the World Medical Association (Declaration of Helsinki). All chemicals, if not stated otherwise, were obtained from Merck Millipore (Bayswater, VIC, Australia).

### Experimental Protocol

The present study was conducted as a series of interconnected experiments to address the primary aims. Experiment One was designed to interrogate the mechanisms of intracellularly generated O_2_^-^ via phenazine methosulfate (PMS; Sigma-Aldrich Pty Ltd, Castle Hill, NSW, Australia) on RBC deformability; specifically the activation state of RBC-NOS and the activation of an important regulatory protein upstream (i.e., PI3K/Akt kinase) were examined. Experiment Two was subsequently designed to examine whether a known positive stimulator of RBC-NOS activation – shear stress – was able to reverse inactivation of RBC-NOS by O_2_^-^, and subsequently restore native cell deformability. Given the high reactivity of NO and O_2_^-^ toward each other, the antioxidant compound Tiron was used as a positive control to elucidate whether shear stress-mediated effects were mimicking antioxidant effects.

#### Overview of Experiment One

Red blood cells were separated from the plasma phase (1500 × *g* for 10 min), and the buffy coat (i.e., leukocytes and platelets) was removed. The RBC pellet was subsequently washed with isotonic phosphate buffered saline (PBS; pH 7.4) twice, before being resuspended in 1% bovine serum albumin (Sigma-Aldrich Pty Ltd, Castle Hill, NSW, Australia) and PBS to create a 0.4 L⋅L^-1^ hematocrit solution. Cell suspensions were subsequently incubated for 60 min at 37°C with either 50 μmol⋅L^-1^ PMS, or PBS (as control). Immediately following incubation, each sample was centrifuged at 1500 x *g* for 5 min and washed twice with PBS. Samples were subsequently aliquot for measurement of cell deformability, and concurrently fixed in PFA for immunohistochemical analysis. The remaining sample was stored at -80°C until subsequent quantification of intracellular free radical concentrations.

#### Overview of Experiment Two

To determine whether shear conditioning could reverse the impairments in cellular deformability induced by O_2_^-^, and provide insights into the underlying mechanism(s), the same O_2_^-^ generating protocol was conducted as described for Experiment One; however, following incubation with/out PMS, cell suspensions were then exposed to 300 s of shear stress (5 or 20 Pa). An O_2_^-^ scavenger (1 mmol⋅L^-1^ 4,5-dihydroxy-1,3-benzene disulfonic acid, “Tiron;” Sigma-Aldrich Pty Ltd, Castle Hill, NSW, Australia) was also introduced in the experiment for comparative purposes with the mechanical stimulation trials; a 10 minute pre-incubation with Tiron was performed in this subset to allow intracellular accumulation of the antioxidant. Immediately following shear exposure, samples were aliquot for measurement of cell deformability, and concurrently fixed in PFA for subsequent immunohistochemical analysis.

### Shear Conditioning of Blood Samples

Cell suspensions were diluted in isotonic polyvinylpyrrolidone solution (30 ± 0.5 mPa⋅s; 7.4 ± 0.5 pH; 290 ± 5 mOsm⋅kg^-1^), and exposed to either 5 or 20 Pa shear stress for 300 s, using a cup-and-bob Couette shearing system (Mechatronics, Hoorn, Netherlands). Cells were inserted into a 300 μm gap between the cylinders; the outer cylinder (cup) rotated around the inner cylinder (bob) at a discrete velocity (shear rate), which was adjusted to generate the desired shear stress. The selected combinations of shear stress-exposure duration have been previously reported to consistently trigger a significant improvement in cellular deformability (Kuck et al., 2018); these were confirmed in the present study during pilot testing. Samples sheared using the cup-and-bob system were analyzed immediately for cellular deformability. Experiments were repeated in a cone-plate viscometer using blood adjusted to 0.4 L⋅L^-1^ hematocrit to confirm findings, and to increase the cell number for immunohistochemistry assays. In these confirmatory studies, whole blood samples were exposed to 0.5 Pa to compare with an earlier report (Ulker et al., 2009). Upon completion, RBC were collected from the viscometer and immediately fixed using 4% PFA for subsequent immunohistochemical procedures.

### RBC Deformability Measurement

Red blood cells deformability was measured using a laser diffraction ektacytometer (Laser Optical Rotational Cell Analyzer, MaxSis, Mechatronics, Hoorn, Netherlands). The details of this device have been described in detail (Hardeman et al., 2001). In brief, the system consists of a co-axial, couette cylindrical shearing system. Two cylinders (300 μm gap) provide a reservoir for cell suspensions (1 mL; RBC in polyvinylpyrrolidone; 30 ± 0.5 mPa⋅s; 7.4 ± 0.5 pH; 290 ± 5 mOsm⋅kg^-1^) that may be sheared and analyzed. The inner cylinder is static, and the outer cylinder rotates at a desired velocity to induce shear. A laser (670 nm; <5 mW) is housed within the inner cylinder, which projects through the blood suspension and generates a diffraction pattern that reflects the average cell morphology in the sample. The diffraction patterns are analyzed in real-time to determine cell deformation. RBC were sheared at ten discrete shear stresses over the range 0.3–30.0 Pa such that an ellipse could be fit to the resultant laser diffraction pattern; diffraction patterns are circular for cells at stasis/under low shears, and become progressively elliptical under higher shears. An elongation index (EI) was calculated at each shear stress using the long and short axes of the ellipse (*a* and *b*, respectively) using the equation: EI = (*a-b*)*/*(*a*+*b*). The EI-shear stress curves were parameterized using a non-linear version of the Lineweaver–Burk equation, as described by Baskurt et al. (2009), yielding the theoretical maximal elongation at infinite shear stress (i.e., EI_max_) and the shear stress necessary to induce half-maximal elongation (i.e., SS_1/2_). Increased EI_max_, and decreased SS_1/2_, reflect improved deformability, and vice versa. SS_1/2_ was also expressed relative to EI_max_ to normalize for changes in maximal deformability; higher/lower values of SS_1/2_:EI_max_ reflect worse/better deformability. All measurements were conducted at 37 ± 0.2°C.

### Immunohistochemical Procedure

Red blood cells were fixed in 4% PFA as described earlier (Suhr et al., 2012) and blood smears were prepared. The smears were heat-fixed using a laboratory gas burner and left to dry overnight. A hydrophobic pen was used to create a test region and control region on each slide. The cells in both areas were washed with tris-buffered saline (0.1 mol⋅L^-1^, pH 7.6) twice, before being incubated with 0.1% Trypsin solution for 30 min at 37°C to permeabilize the cell membrane. Cells were then incubated in solution (2% hydrogen peroxide; 80% methanol) for 30 min at room temperature, followed by a 30 min exposure (room temperature) to 3% skim milk in tris-buffered saline to minimize non-specific binding. The test field of each slide was incubated with the respective primary antibody (i.e., Phospho-eNOS^Ser1177^ or Phospho-Akt^Ser473^; Cell Signaling, MA, United States) diluted at 1:150 in a 0.3% skim milk solution for 60 min at room temperature; the control field on each slide was treated identically, except that it was not incubated with the primary antibody during this step. Unbound primary antibody was then washed off the slides using tris-buffered saline, prior to blocking with 3% normal goat serum. Cells were then incubated with a secondary antibody (goat anti-rabbit; dilution: 1:400, Dako, Glostrup, Denmark) for 30 min at room temperature. In order to develop staining, a 3,3-diaminobenzidine-tetrahydrochloride solution (Sigma, St. Louis, MO, United States) in 0.1 mol⋅L^-1^ tris-buffered saline was utilized. Slides were then dehydrated by exposure to alcohol solutions of increasing concentration and sealed using a purpose mounting medium. Images of the cells were acquired using an inverted microscope (IX73, Olympus, Tokyo, Japan) and integrated camera (optiMOS sCMOS, QImaging, Surrey, SA, Australia) at 400-fold magnification and analyzed with open source software (Image J, National Institutes of Health, Bethesda, MD, United States). At least 100 individual RBC per test area, and 50 cells per control area, from at least 2 different regions on each slide, were randomly chosen and respective gray values were determined. The gray values were averaged before the background value of each area was measured and subtracted from the former value. The control area value was subtracted from the test area value to determine the final signal.

### Free ROS Level

A commercial immunoassay (OxiSelect In Vitro ROS/RNS Assay Kit, Cell Biolabs Inc., San Diego, CA, United States) was used to estimate free radical concentrations in RBC as previously described (Grau et al., 2016). This assay employs a proprietary quenched fluorogenic probe, dichlorodihydrofluorescin (DCFH) DiOxyQ, which is a specific probe for reactive oxygen and nitrogen species (i.e., ROS and RNS). The DCFH-DiOxyQ probe was first processed to receive a highly reactive DCFH form. ROS in the sample may react with DCFH, which is rapidly oxidized to the highly fluorescent 2′,7′-dichlorodihydrofluorescein form. The prepared dichlorodihydrofluorescin probe was added to lysed RBC and standards, and the reaction was allowed to proceed for 45 min at room temperature. Fluorescence was read with a fluorescence plate reader (Fluoroskan Ascent Microplate Fluorometer; Thermo Fisher Scientific) at 480 nm excitation and 530 nm emission. Samples were then measured against a dichlorodihydrofluorescin standard and concentration was calculated by linear regression.

### Statistical Analysis

All results are presented as mean ± standard error. Raw RBC elongation indexes were compared utilizing a two-way analysis of variance, while curve-fit parameters (i.e., EI_max_, SS_1/2_) were compared using one-way analysis of variance to detect significant differences in mean values. To compare protein phosphorylation pre- and post-intervention, paired *t*-tests were employed. Statistical analyses were conducted using commercial software (Prism, GraphPad Software Inc., La Jolla, CA, United States) using an alpha level of 0.05.

## Results

### Experiment 1: Effect of Free Radical Production on RBC Physiology

#### Free Reactive Species

**Figure 1:**
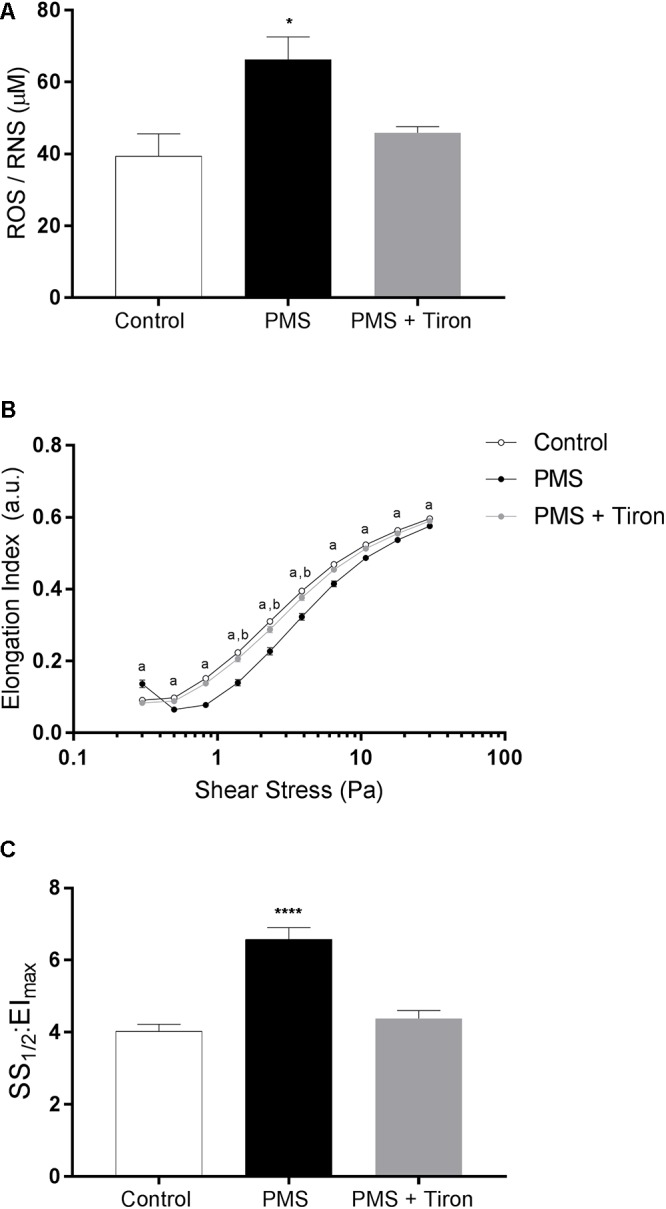
The concentration of total reactive oxygen species (ROS) increased in phenazine methosulfate-treated cells (PMS) compared to control red blood cells (RBC; **A**). Concurrent incubation with PMS and Tiron decreased total ROS to Control levels. RBC elongation indices (EI), quantified over a range of shear stresses (0.3–30 Pa), were decreased in PMS-treated RBC when compared to control; Tiron partially prevented impaired deformability **(B)**. A global parameter of cell deformability was also impaired in PMS-treated RBC compared to control **(C)**, although Tiron reversed this effect. ^a^Control significantly different to PMS. ^b^Control different compared with PMS + Tiron. ^∗∗∗∗^*p* < 0.0001 significantly different to Control. ^∗^*p* < 0.05 significantly different compared to Control.

The concentration of total free radical species (i.e., ROS and RNS) following incubation with (PMS) and without (Control) a O_2_^-^ generating agent, and concurrent incubation with PMS and the antioxidant Tiron, is presented in Figure 1A. Incubation with PMS significantly increased intracellular ROS/RNS in RBC by ∼64%, when compared with Control (*p* < 0.05). Addition of Tiron to PMS-incubated RBC significantly decreased ROS/RNS concentrations, which were not different to Control levels (*p* = 0.981).

#### RBC Deformability Under Oxidative Stress

The cellular deformability of RBC exposed to PMS, PMS and Tiron, or PBS (control) is presented in Figure 1B,C. Incubation with PMS resulted in a significant “right-shift” in the EI-shear stress response, indicative of impaired cellular deformability, at all measured shears (i.e., 0.3–30 Pa; *p* < 0.0001). This impaired deformability response was most pronounced at 1∼10 Pa. An atypical increase in EI of PMS-treated cell suspensions was noticed at the lowest shear stress employed (i.e., 0.3 Pa). The index that reflects global cellular deformability (i.e., SS_1/2_:EI_max_) supported that cell deformability was impaired by PMS, given SS_1/2_: EI_max_ increased (i.e., was impaired) by ∼61% (*p* < 0.0001), whereas the addition of Tiron diminished this impairment.

#### Free Radical Effects on Activation States of RBC-NOS and Akt

**Figure 2:**
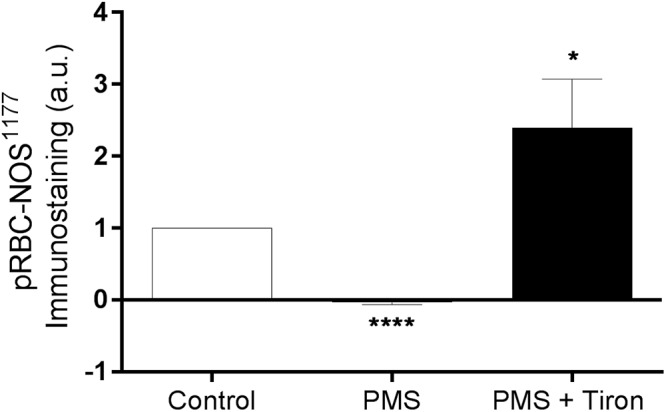
Relative density of immunostained RBC treated with primary antibody against phosphorylated RBC-NOS Ser^1177^; RBC incubated with the phenazine methosulfate (PMS) and the addition of a superoxide scavenger (Tiron) are reported as relative changes compared with Control RBC. ^∗∗∗∗^*p* < 0.0001 significantly different compared with Control, ^∗^*p* < 0.05 significantly different compared with Control. Error bars absent for Control, due to relative measurement.

**Figure 3:**
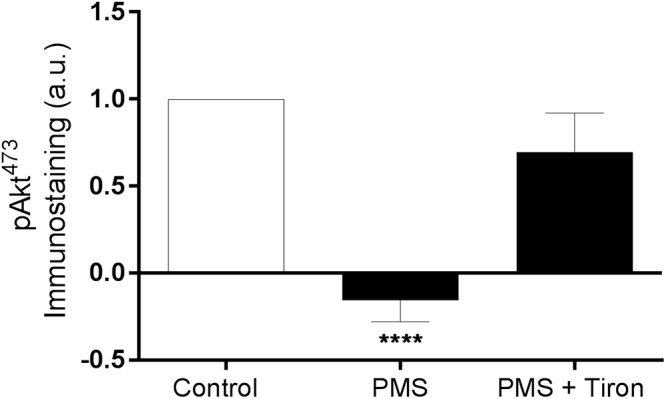
Relative density of immunostained RBC treated for phosphorylated Akt Ser^473^; RBC incubated with phenazine methosulfate (PMS) and the addition of a superoxide scavenger (Tiron) are reported as relative change compared with Control. ^∗∗∗∗^*p* < 0.0001 significantly different compared with Control. Error bars absent for Control, due to relative measurement.

The active site of RBC-NOS (i.e., Serine 1177) was examined for RBC following exposure to PMS, when compared with Control (Figure 2). Incubation of RBC with PMS resulted in inactivation of RBC-NOS (i.e., 0% phosphorylated RBC-NOS Ser^1177^; *p* < 0.05) (Figure 2). Immunohistochemical staining of phosphorylated protein kinase B (i.e., Akt kinase) at serine residue 473 (i.e., pAkt Ser^473^) demonstrated that PMS-treatment reduced Akt to an inactive state (i.e., 0% activity; Figure 3). Co-incubation of RBC with PMS and Tiron, however, prevented ∼70% of the observed reduction in phosphorylated Akt^473^ (*p* < 0.01; Figure 3).

### Experiment 2: Reversibility of O_2_^-^-Induced Impairments to RBC

#### Shear-Mediated Augmentation of RBC Deformability

**Figure 4:**
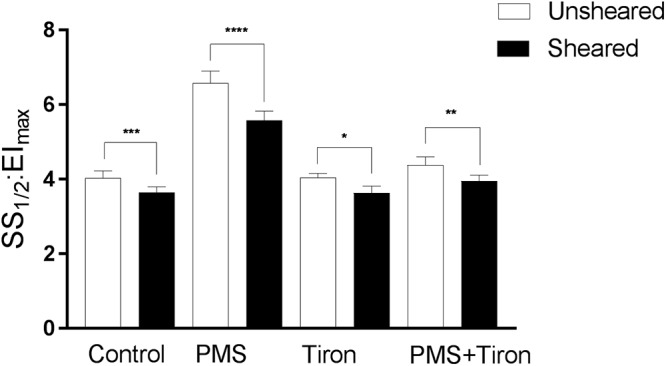
Cellular deformability (expressed as SS_1/2_:EI_max_) of RBC exposed to phenazine methosulphate, a superoxide scavenger (Tiron), or combined PMS and Tiron (PMS + Tiron). Data collected for cells following 60 min “rest” (unsheared) or following 5 min of shear conditioning (sheared; see text for details). Significantly different between designated pairing at ^∗∗∗^*p* < 0.001, ^∗∗^*p* < 0.01, or ^∗^*p* < 0.05.

To determine whether shear conditioning could reverse PMS-induced impairments in RBC physiology, combined mechanical stress and oxidative stress were examined. The effect of shear conditioning significantly modulated the RBC deformability response even in PMS-treated RBC (Figure 4); that is, shear conditioning caused a left-shift (i.e., improvement) of the EI-shear stress curves independent of the presence of O_2_^-^. Specifically, Control RBC that were exposed to 20 Pa for 300 s in the absence of PMS demonstrated an improvement in cellular deformability, as manifested by an increase of EI when measured at 0.83 and 1.39 Pa (data not shown). This was confirmed when exploring a global parameter of RBC deformability, where shear conditioning using 20 Pa for 300 s significantly decreased SS_1/2_:EI_max_ (i.e., improved cell deformability) of Control cells by ∼11% (*p* < 0.01). Exposure to 20 Pa for 300 s also induced an improvement in cell deformability for RBC previously exposed to PMS, as manifested by an increase in the EI when measured at a wide range of shears (0.5–3.87 Pa, *p* < 0.05). This shear-induced improvement in cell deformability of PMS-treated cells was reflected by an ∼18% decrease in SS_1/2_:EI_max_ (*p* < 0.0001).

#### Shear Conditioning Reversed Impairments to RBC-NOS

**Figure 5:**
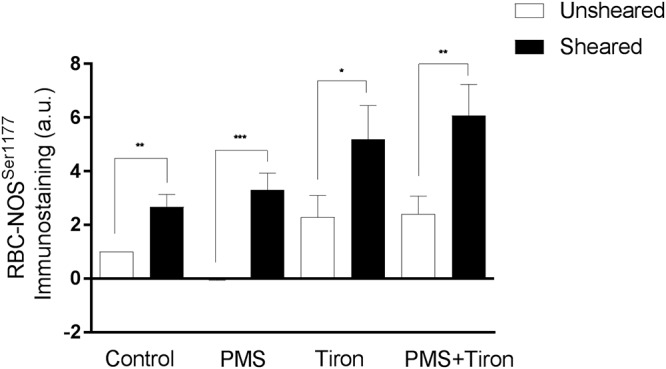
Relative density of immunostained RBC for RBC-NOS Ser^1177^; results reported as relative change in density compared to Control RBC. Significantly different between designated pairing at ^∗∗∗^*p* < 0.001, ^∗∗^*p* < 0.01, or ^∗^*p* < 0.05.

The active site of RBC-NOS (i.e., Serine 1177) was examined for RBC previously exposed to shear stress, and combined shear and PMS, and compared with Control (Figure 5). Shear stress exposure significantly increased phosphorylation of RBC-NOS^1177^ compared with Control (*p* < 0.05). When RBC were incubated with PMS, subsequent exposure to shear stress restored RBC-NOS^1177^ activation (*p* < 0.01) to levels comparable with Control cells that had been exposed to shear. Further experiments were performed, confirming that the effects of mechanical stimulation on RBC-NOS^1177^ were congruent when the shear duration-magnitude combinations utilized in ektacytometry and immunohistochemistry measurements were matched (i.e., 5 Pa for 300 s; Supplementary Figure 1).

#### Antioxidant Treatment Reversed PMS-Induced Impaired RBC Deformability

Cellular deformability expressed as a single parameter (i.e., SS_1/2_:EI_max_) is illustrated in Figure 4. Tiron incubation in isolation did not affect RBC deformability. Upon the addition of PMS, however, Tiron-treatment was effective at decreasing the impairment of cell deformability induced by free radicals, reflected by a significant decrease of SS_1/2_:EI_max_ by ∼32.5% (*p* < 0.001; Figure 4) relative to PMS-incubated cells. Moreover, shear conditioning of these samples resulted in a further decrease (i.e., improvement) of SS_1/2_:EI_max_ by ∼9 and ∼12% using 5 and 20 Pa shear stress, respectively.

#### Antioxidants Augmented RBC-NOS and Akt Activation

Application of Tiron, a vitamin E analog, induced an increase in basal phosphorylation (relative to Control RBC phosphorylation levels; Figure 5). Parallel to the increased phosphorylated RBC-NOS Ser^1177^ at baseline, shear conditioning resulted in a 2.3-fold increase of phosphorylated Ser^1177^ in Tiron-treated RBC (*p* < 0.05) compared to sheared Control RBC. When Tiron-treated RBC were concurrently exposed to PMS, baseline phosphorylation was unaffected; however, shear conditioning resulted in a 2.5-fold increase (*p* < 0.01). Of note, in both Control and Tiron-treated RBC, the relative increase was more pronounced in the presence of the O_2_*^-^* generation by PMS. The application of Tiron also resulted in partial restoration of PMS-induced impaired pAkt Ser^473^ activation by 70% (*p* < 0.01; Figure 3).

## Discussion

The salient findings of the present study were that conditioning shear exposure within the physiological range (i.e., 5–20 Pa) improves RBC deformability despite the confirmed presence of elevated levels of free radicals (Figure 1). Moreover, we report that increased RBC-NOS activity, induced by physiological levels of shear conditioning, occurred in tandem with improved RBC deformability. O_2_^-^ generation within RBC caused impaired cell deformability, confirming previous reports (Baskurt et al., 1998), which occurred concomitantly with a novel observation that RBC-NOS was inactivated (i.e., phosphorylated RBC-NOS Ser^1177^ 0%; Figure 2). When a O_2_^-^-scavenging antioxidant compound was applied, the negative effects of O_2_^-^ generation were partly diminished; however, shear conditioning was still effective at improving RBC deformability (Figure 5), indicating that mechanical stimulation may increase cellular deformability via a mechanism that is independent of O_2_^-^ scavenging. Collectively, our data indicate that conditioning RBC with physiological shear stress improves cell deformability irrespective of oxidative stress, which is partly mediated by increased RBC-NOS activity. The current work highlights a complex relationship between mechanical and oxidative stresses within RBC physiology.

In the present study, exposure to O_2_^-^ significantly impaired RBC deformability. This observation agrees with previous work, in which the authors proposed protein cross-linking as the primary mechanism prompting impaired cell deformability, despite not detecting differences in RBC membrane proteins by gel electrophoresis (Baskurt et al., 1998; Simmonds et al., 2011). We support these findings and further propose that intracellular signaling pathways known to be involved in the regulation of cell deformability may be disrupted by intracellular free radicals, thus impairing cell deformability under oxidative stress. The results of the present indicate that phosphorylation of RBC-NOS at Ser^1177^, which is recognized to regulate NOS-activation (Crabtree and Channon, 2011) and influence cell deformability, was reduced to 0% (i.e., no enzymatic NO-production) in the presence of O_2_^-^. Tetrahydrobiopterin (BH_4_), an essential cofactor for NOS-related NO-production, may rapidly react with O_2_^-^, forming BH_2_ (Crabtree and Channon, 2011). When BH_4_-availability is reduced, the NOS enzyme is unable to transfer electrons to the N-terminal oxygenase domain, which results in NOS generating O_2_^-^ rather than NO (Luo et al., 2014). Given phosphorylation of the active site of RBC-NOS was impaired (Figure 2), however, the current results indicate that rather than uncoupling of RBC-NOS by reduced BH4 availability, O_2_^-^ directly prevents phosphorylation of RBC-NOS, resulting in decreased overall activity of the enzyme and thus decreased NO generation.

Reduced NO bioavailability is typically associated with impaired cell deformability, while increased intracellular NO availability augments cell deformability (Bor-Kucukatay et al., 2003), likely due to covalent binding of cytoskeletal proteins such as spectrin (Grau et al., 2013). The impaired RBC deformability observed in PMS treated RBC (Figure 1B,C) may reflect a reduced intracellular availability of NO, possibly due to O_2_^-^ quenching of NO. Intracellular production of O_2_^-^ may facilitate formation of ONOO^-^ by reacting with NO, including from sources independent of RBC-NOS (e.g., nitrite reduction via Hb). ONOO^-^ is well-known to induce cytotoxic effects; that is, ONOO^-^ mediates protein modifications by forming nitrotyrosine residues. Moreover, ONOO^-^ significantly impairs Akt activity in endothelial cells (Zou et al., 2002). While preliminary studies indicate that nitrotyrosine-formation may be increased in RBC exposed to intracellular O_2_^-^ (data not shown), further studies investigating nitrotyrosine formation as a marker for ONOO^-^ generation in RBC may be of value to clarify the potential involvement of other nitrogen radicals.

It is possible that free radical-mediated interference in the signaling pathway upstream of RBC-NOS may be involved in O_2_^-^-mediated inactivation of this protein; in healthy RBC, RBC-NOS activation requires phosphorylation of residue Ser^1177^. Protein kinase B (Akt) acts to phosphorylate RBC-NOS; Akt itself is activated by phosphorylation of Ser^473^ via PI3K upstream. We observed that PMS treatment significantly inhibited phosphorylation of Akt^473^ (Figure 3), indicating that oxidative stress may interfere with the Akt/PI3K signaling pathway in RBC. Application of a O_2_^-^ scavenger (i.e., Tiron) prevented inhibition of phosphorylated Akt^473^ (Figure 3), confirming that O_2_^-^ was involved. Although studies of the PI3K/Akt signaling pathway within RBC are sparse, elevated O_2_^-^ levels have been associated with decreased phosphorylation of Akt in myocytes of heart failure patients – a pathology characterized by oxidative stress (Ohta et al., 2011). Accumulating evidence suggests that oxidation of methionine residues by reactive oxygen species, such as O_2_^-^, may influence and inhibit phosphorylation of serine residues in kinases as a protective mechanism in states of oxidative stress (Rao et al., 2015). Recent proteomic analyses demonstrate that methionine residues in proximity to phosphorylation target sites are preferred targets of oxidation, leading to the proposal that potential cross-talk exists between these two mechanisms of post-translational modifications (Veredas et al., 2017). Although further studies are necessary to confirm whether this hypothesis applies to RBC, it is possible that inhibited phosphorylation of active sites in both RBC-NOS and Akt kinase is caused by oxidation of nearby methionine residues. Moreover, given the range of active proteins in RBC regulated by post-translational modification (Pasini et al., 2006), it is possible that intracellular free radicals may alter a broad variety of proteins beyond the Akt/RBC-NOS pathway. Free radicals are also highly reactive toward biomolecules other than proteins, including lipids and nucleotides; thus, additional detrimental effects initiated by intracellular free radical generation (e.g., oxidative modifications of membrane proteins/lipids) need to be considered, and are of value for future investigation.

The collective results of Experiment One indicate that intracellular O_2_^-^ generation results in impaired RBC deformability, which may be explained by inhibited phosphorylation of serine residues in enzymes that contribute to the regulation of cell deformability (i.e., RBC-NOS and Akt). That an O_2_^-^ scavenger was able to ameliorate the inhibition of the activation sites of these proteins further supports this finding, and may yield valuable therapeutic outcomes.

Experiment Two was designed to interrogate whether physiological magnitudes of shear exposure could recover, atleast in part, some of the impaired mechanical properties of RBC that had been previously exposed to free radicals. It was hypothesized that shear exposure within the physiological range would stimulate RBC-NOS phosphorylation at Ser^1177^, and therefore increase cellular deformability despite the prior oxidative “damage.” Mechanical stimulation of RBC significantly increased cell deformability by ∼18% (*p* < 0.01, Figure 4) and increased phosphorylation of RBC-NOS (*p* < 0.01, Figure 5), confirming previous reports (Ulker et al., 2011; Kuck et al., 2018). The current observations extend these findings, providing evidence that mechanical stimulation of RBC is also effective at improving cellular deformability in cells exposed to O_2_^-^ (Figure 4). This occurred in tandem with re-phosphorylation of RBC-NOS at Ser^1177^, which was inhibited by O_2_^-^ for RBC that were not exposed to shear stress. Although NO concentration was not directly quantified in the present study, Ulker et al. (2011) reported that shear stress and resultant phosphorylation of RBC-NOS mediate increased NO availability within RBC. In the presence of elevated O_2_^-^ levels, however, it is plausible that NO may scavenge O_2_^-^ to form ONOO^-^, given that both molecules are free radicals and thus exhibit extremely rapid (<1 ms) reaction kinetics with each other (Huie and Padmaja, 1993).

To interrogate whether mechanically stimulated NO production and subsequent reaction of NO with O_2_^-^ (i.e., scavenging of free radicals) was responsible for reversing the PMS-induced impairments, we incorporated a O_2_^-^ scavenging, membrane permeable O_2_^-^ scavenger (Tiron) to regulate O_2_^-^. While not affecting basal RBC deformability, application of Tiron significantly decreased impairments induced by PMS incubation (*p* < 0.001; Figure 4). Moreover, incubation of RBC with Tiron significantly increased phosphorylated Ser^1177^ in PMS-treated RBC (*p* < 0.01; Figure 5) to levels comparable with mechanically stimulated Control RBC. Given the congruent effects of mechanical stimulation and application of a O_2_^-^ scavenging agent (i.e., partially restoring cell deformability), these data potentially indicate that mechanical stimulation may increase RBC-NOS mediated NO generation, and NO may act as a O_2_^-^ scavenger. It should be noted, however, that removal of O_2_^-^ by NO yields ONOO^-^, a powerful oxidant with potential to induce distinct adverse effects as mentioned above. On the other hand, it is possible that RBC-NOS generated NO may improve cell mechanics via S-nitrosylation of integral cell membrane proteins (Grau et al., 2013), therefore overcoming some of the O_2_^-^ mediated impairments in cell deformability. Whether mechanically stimulated improvement in RBC mechanics are related to NO scavenging O_2_^-^, or nitrosylation of the cell membrane, or a combination of each of these processes, requires further exploration.

Neither intervention (i.e., mechanical stimulation or antioxidant supplementation) fully restored RBC deformability back to Control levels when applied individually (Figure 4). RBC that were pre-incubated with Tiron prior to PMS exposure exhibited no significant differences in Ser^1177^ at baseline or after shear conditioning, when compared to RBC incubated with Tiron alone (*p* = 0.466; Figure 5). It appears that the beneficial effects of mechanical stimulation on RBC deformability are independent of merely scavenging O_2_^-^. While Tiron was able to eliminate the effects of O_2_^-^ (Figure 4), mechanical stimulation improved cell deformability irrespective of O_2_^-^ presence, and also for cells “normalized” by O_2_^-^ scavenging. Given that the application of Tiron was observed to counteract the effects of O_2_^-^ generation on Ser^1177^ phosphorylation in unsheared RBC (Figure 5), it thus appears that RBC-NOS activation and shear-mediated NO generation improve cell deformability independent of a potential “antioxidant” effect of NO.

Collectively, the salient findings of the present study indicate that O_2_^-^ generation within RBC interferes with molecular pathways involved in regulating cell deformability – i.e., PI3K/Akt and RBC-NOS. Application of the antioxidant compound Tiron, however, restored activation of these enzymes. Moreover, mechanical stimulation utilizing physiological magnitudes of shear stress improved RBC deformability and restored phosphorylation of RBC-NOS Ser^1177^, irrespective of the presence of O_2_^-^ or Tiron via an unresolved mechanism. The potential for improving the physical properties of RBC by mechanical stimulation to increase tissue perfusion is of special interest in therapeutic interventions and surgical procedures.

## Author Contributions

MS, MG, and LK conceptualized, designed, and performed the experiments. MS, MG, LK, and WB analyzed and interpreted the data. MG, WB, and MS provided the reagents. MS, MG, and WB edited and critically reviewed the manuscript. LK and MS wrote the manuscript.

## Conflict of Interest Statement

The authors declare that the research was conducted in the absence of any commercial or financial relationships that could be construed as a potential conflict of interest.
