# Supplementary material for: Shear Stress Ameliorates Superoxide Impairment to Erythrocyte Deformability With Concurrent Nitric Oxide Synthase Activation
Source: Front Physiol. 2019 Feb 5;10:36. doi: 10.3389/fphys.2019.00036 (PMC6370721; doi:10.3389/fphys.2019.00036)
Supplement: Supplementary file 1 [file Image_1.pdf]

Supplementary Material

Shear stress ameliorates superoxide impairment to erythrocyte deformability with concurrent nitric oxide synthase activation

Lennart Kuck, Marijke Grau, Wilhelm Bloch, Michael J. Simmonds*

***Correspondence:** Dr Michael J. Simmonds: mike@thesimmonds.id.au

1.1 Supplementary Figures

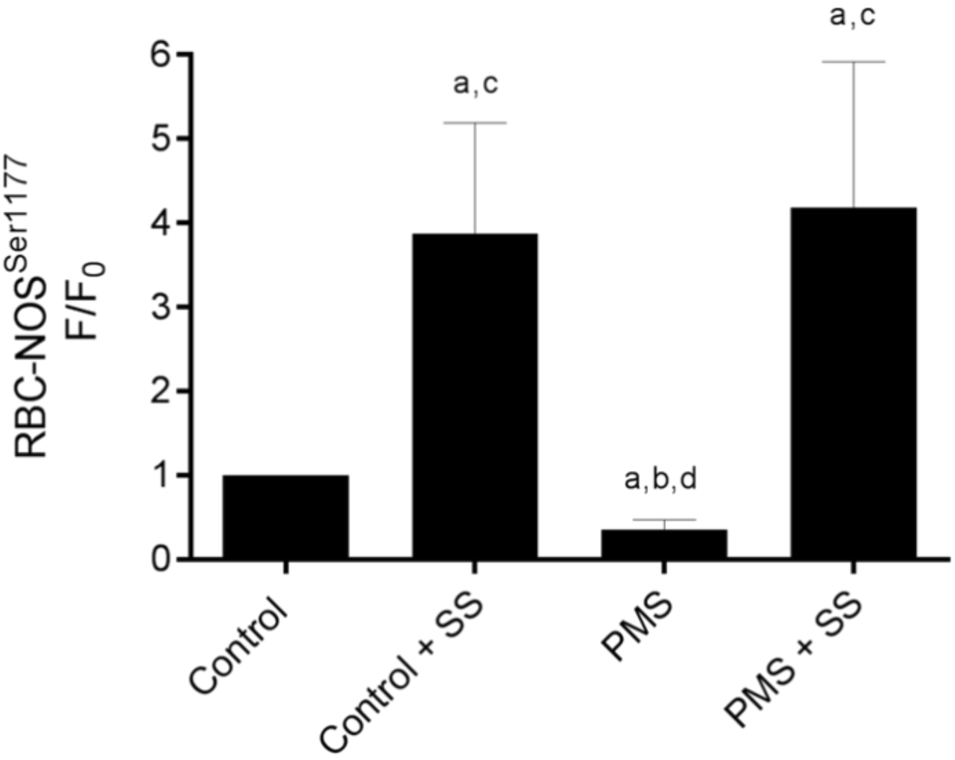

Supplementary Figure 1: Red blood cells (RBC) exposed to shear stress (SS), oxidative stress (PMS), or a combination of the two (PMS + SS). Data (n = 8) presented as relative change compared with Control (i.e., Control = 1.0, thus no error bars for Control). Immunofluorescence of RBC exposed to shear conditioning (i.e., 5 Pa for 5 min), matching the shear regime employed to obtain cell deformability data. [a], significantly different from Control. [b], significantly different from Control + SS. [c], significantly different from PMS, [d], significantly different from PMS + SS.
